# Revisiting Vaccine Hesitancy in Residential Care Homes for the Elderly for Pandemic Preparedness: A Lesson from COVID-19

**DOI:** 10.3390/vaccines11111700

**Published:** 2023-11-08

**Authors:** Cyrus Lap Kwan Leung, Wan In Wei, Kin-Kit Li, Edward B. McNeil, Arthur Tang, Samuel Yeung Shan Wong, Kin On Kwok

**Affiliations:** 1 JC School of Public Health and Primary Care, The Chinese University of Hong Kong, Hong Kong, China; cyrusleung@cuhk.edu.hk (C.L.K.L.); vivian1628@cuhk.edu.hk (W.I.W.); emcneil@cuhk.edu.hk (E.B.M.); yeungshanwong@cuhk.edu.hk (S.Y.S.W.); 2 Department of Social and Behavioural Sciences, City University of Hong Kong, Hong Kong, China; ben.li@cityu.edu.hk; 3 School of Science, Engineering and Technology, RMIT University, Ho Chi Minh City 700000, Vietnam; arthur.tang@rmit.edu.vn; 4 Stanley Ho Centre for Emerging Infectious Diseases, The Chinese University of Hong Kong, Hong Kong, China; 5 Hong Kong Institute of Asia-Pacific Studies, The Chinese University of Hong Kong, Hong Kong, China

**Keywords:** vaccine hesitancy, loneliness, residential care homes for the elderly, disease transmission, infectious, prevention and control, COVID-19

## Abstract

Residents in residential care homes for the elderly (RCHEs) are at high risk of severe illnesses and mortality, while staff have high exposure to intimate care activities. Addressing vaccine hesitancy is crucial to safeguard vaccine uptake in this vulnerable setting, especially amid a pandemic. In response to this, we conducted a cross-sectional survey to measure the level of vaccine hesitancy and to examine its associated factors among residents and staff in RCHEs in Hong Kong. We recruited residents and staff from 31 RCHEs in July–November 2022. Of 204 residents, 9.8% had a higher level of vaccine hesitancy (scored ≥ 4 out of 7, mean = 2.44). Around 7% of the staff (*n* = 168) showed higher vaccine hesitancy (mean = 2.45). From multi-level regression analyses, higher social loneliness, higher anxiety, poorer cognitive ability, being vaccinated with fewer doses, and lower institutional vaccination rates predicted residents’ vaccine hesitancy. Similarly, higher emotional loneliness, higher anxiety, being vaccinated with fewer doses, and working in larger RCHEs predicted staff’s vaccine hesitancy. Although the reliance on self-report data and convenience sampling may hamper the generalizability of the results, this study highlighted the importance of addressing the loneliness of residents and staff in RCHEs to combat vaccine hesitancy. Innovative and technology-aided interventions are needed to build social support and ensure social interactions among the residents and staff, especially amid outbreaks.

## 1. Introduction

### 1.1. An Outbreak-Prone Healthcare Setting

Residential care homes for the elderly (RCHE) are a vulnerable healthcare setting characterized by the frailty of RCHE residents (“residents”), inevitable close contact between RCHE staff (“staff”) and residents, and the congregate living environment. These characteristics render them prone to outbreaks of infectious diseases. A recent prominent outbreak was caused by COVID-19 [1]. The devastating impact of COVID-19 on RCHEs is portrayed as a “perfect storm” [2,3], as reflected by a high incidence and fatality rate among the residents. Though social distancing measures, such as the suspension of group activities, were implemented, SARS-CoV-2 still went viral.

### 1.2. Vaccine Hesitancy before Explosive Omicron Outbreaks in RCHEs

Vaccination is an essential infection control measure, but there has been vaccine hesitancy in RCHEs. While Western countries saw successful vaccination efforts, East and Southeast Asia struggled with widespread COVID-19 vaccination. In late 2021, 58.1% of RCHE residents were vaccine hesitant in China [4]. In Hong Kong, before the explosive fifth Omicron-dominant wave in early 2022, the proportion of individuals aged 80 years or above completing a two-dose primary vaccination series was low (18%) [5]. Complex decision-making processes for vaccinations among older adults were impacted by trust, social support networks, and cultural stereotypes [6]. For RCHE residents, other factors were notable in predicting vaccine hesitancy, including symptoms of dementia [7] and confidence in the safety of vaccines [8].

### 1.3. The Need to Revisit Vaccine Hesitancy in RCHEs after Explosive Omicron Outbreaks

Understanding the factors contributing to vaccine hesitancy in RCHEs is imperative for better preparedness for future outbreaks. There are several reasons to revisit this topic after the explosive Omicron outbreaks.

First, vaccine hesitancy is mutable [9], and is perhaps sensitive to previous disease exposure as in the case of non-pharmaceutical interventions [10]. With RCHEs hit hard in the COVID-19 pandemic [1], witnessing severe infections and deaths of their peers heightened the risk perceptions of residents and staff [11], which might induce changes to vaccine hesitancy in RCHEs.

Second, during COVID-19, there was a prolonged visit ban imposed on RCHEs [12], which socially isolated residents, in turn leading to the deprivation of social support or social loneliness [13]. Furthermore, the detachment from loved ones resulted in emotional loneliness [14]. The pandemic and social distancing measures increased staff workload [15] and deprived them of social and emotional support [16]. Social interaction and support provide crucial information and unease with the feeling of uncertainty in urban disasters [17], and lacking such information and support would influence preparedness behavior, including vaccination behavior, e.g., [18].

Third, vaccine mandates, as in the case of COVID-19, may not be ethical [19]. Vaccine mandates without adequate societal support and confidence might trigger psychological reactance and societal backlash [20], decreased support of vaccination in general [21], and demotivation of active vaccination behavior [22]. Coercion that is inconsistent with the residents’ free will could be detrimental to their mental health. Therefore, resolving vaccine hesitancy is of utmost public health concern as it avoids vaccine mandates in the future.

Fourth, the attitudes of healthcare providers played an important role in shaping the vaccine hesitancy of the residents. Healthcare providers remained the most trusted sources for healthcare advice [9], and, at the same time, they could transmit diseases to residents (and vice versa) [23,24]. One study quantified that the rate of COVID-19 among residents could be lowered by 2% per percentage increase in staff vaccine uptake [25]. It is therefore indispensable to address vaccine hesitancy among staff.

### 1.4. Study Aims and Significance

Investigating vaccine hesitancy of both staff and residents is informative for designing holistic interventions for future outbreaks. Therefore, we aim to measure the level of vaccine hesitancy and to examine its contributing factors among residents and staff shortly after explosive Omicron outbreaks in RCHEs.

## 2. Methodology

### 2.1. Study Design

We conducted a cross-sectional survey among residents and staff from licensed RCHEs in Hong Kong. Invitations were sent to administrators of all RCHEs, who were approached one by one until the sample size was reached. Enrolled RCHEs were asked to provide a list of residents who were able to consent on their own or whose guardian consent was obtainable. The research team visited the study RCHEs in February–November 2022, and the survey period was July–November 2022.

All consenting staff were eligible to participate, while only consenting residents who were cognitively capable could participate. To ensure valid self-report responses, the research team first assessed residents’ cognitive ability using the Mini-cog [26], a brief screening test with three-item recall and clock drawing. Residents who scored ≥ 3 (out of 5) in the Mini-cog were regarded as cognitively capable.

### 2.2. The Study Instrument for Residents

There are two parts to the study instrument for residents.

Part 1 was completed by interviewing staff in charge or by data retrieval from the RCHE records. Solicited information included demographics, medical history (including COVID-19 vaccination and infection), care level required (low/middle/high), and the degree of functional disability. In particular, functional disability was measured by the Barthel Index [27], which assessed the individual’s ability to perform ten basic activities of daily living. Each activity was scored on a scale, with the total score ranging from 0 (complete dependence) to 100 (complete independence).

Part 2 was self-reported by residents through face-to-face interviews. Solicited information included scales measuring vaccine hesitancy (Section 2.2.1), anxiety (Section 2.2.2), and loneliness (Section 2.2.3).

#### 2.2.1. Vaccine Hesitancy

We used the short version of the 5C psychological antecedents to vaccination [28] to measure vaccine hesitancy. Each 5C construct (confidence, complacency, calculation, constraints, collective responsibility) was measured by a single item in a seven-point Likert scale. We computed a vaccine hesitancy index by adding up the confidence, complacency, and collective responsibility scores with the confidence score reversed (but not the collective responsibility as it is originally a reversed item). We excluded calculation and constraints in the computation as a constraint is a less relevant construct in RCHEs, while the association between calculation and vaccination intention is equivocal, e.g., [29]. A higher index indicated stronger vaccine hesitancy.

#### 2.2.2. Anxiety

We measured the anxiety symptoms using the Long-term Care version of the Geriatric Anxiety Scale (GAS-LTC) [30]. GAS-LTC shares the same items as the 10-item GAS [31] with the response format simplified to “Yes–No”. Participants responded to a series of statements about their feelings of worry, fear, or anxiety, in the past week. A sample item was “I felt like something terrible was going to happen to me.” For the translation of statements, we adopted the Chinese version of the GAS [32].

#### 2.2.3. Loneliness

We used the Cantonese version of the De Jong Gierveld Loneliness Scale [33] to measure the level of loneliness. The scale included items that assessed both emotional loneliness (e.g., “I experience a general sense of emptiness”) and social loneliness (e.g., “There are many people I can trust completely”). Respondents were asked to indicate whether they agreed on a series of statements. The scale was internationally validated in Chinese populations in other countries, e.g., [34,35].

### 2.3. The Study Instrument for Staff

Staff completed the study instrument by themselves. Solicited items included demographics, medical history (including COVID-19 vaccination and infection), vaccine hesitancy (Section 2.3.1), loneliness (Section 2.2.3), and anxiety (Section 2.3.2).

#### 2.3.1. Vaccine Hesitancy

We used the full version of the 5C psychological antecedents to vaccination [28], as we previously did for a community cohort [36]. There are three items for each 5C construct measured in a seven-point Likert scale. Confidence, complacency, and collective responsibility were used in computing the overall vaccine hesitancy index.

#### 2.3.2. Anxiety

We used the 7-item anxiety subscale of the Hospital Anxiety and Depression Scale (HADS) [37] for measurement. Participants rated the statements on a 4-point Likert scale from 0 to 3. Higher scores indicate more symptoms.

### 2.4. The Study Instrument for Facility-Level Attributes

Facility-level attributes of the study RCHEs were collected with a questionnaire completed by the staff in charge of the RCHEs (or their delegate). Solicited items included facility size and geographical regions.

### 2.5. Statistical Analysis

We summarized the demographic characteristics and measurement responses using descriptive statistics. Data with missing values in vaccine hesitancy were discarded from the analyses (*n* = 10). The primary outcomes were the overall vaccine hesitancy index and the individual 5C scores. We identified the associated individual factors of vaccine hesitancy among the residents and staff as first-level factors using multilevel regression analyses (Model 1), with the effects of institutional factors also modeled as the second-level factors (Model 2). Variables included in the multilevel models are in Table 1. A statistical significance of 0.05 was specified. All analyses were conducted in R version 4.3.1.

### 2.6. Ethics Statement

This study was approved by the Joint CUHK-NTEC Clinical Research Ethics Committee (reference number: 2021.643).

## 3. Results

### 3.1. Characteristics of the Residents

There were 204 eligible residents from 28 RCHEs included in the analysis (Table 2). Participating residents consisted of more males (65.7%) with a mean age of 73.00 years (standard deviation (SD) = 12.66). Most reported having chronic diseases (87.3%) and were infected by COVID-19 at least once (85.3%). The majority of the participating residents were vaccinated with at least one dose of the COVID-19 vaccine (97.5%), and more than half of them had taken three doses or more at the time of the study (52.5%). Regarding care level, around half of the residents required middle or high-level care to facilitate their daily instrumental activities, with 49.5% scoring 90 or lower on the Barthel Index.

Vaccine hesitancy among residents was low (mean = 2.44, SD = 1.05) (Table 3). Around 10% of the residents had a higher level of vaccine hesitancy (scored 4 or 4+ out of 7). Institutional mean scores of vaccine hesitancy ranged from 1.55 to 3.08.

### 3.2. Characteristics of the Staff

There were 168 staff from 26 RCHEs enrolled in this study (Table 2). The majority were female (92.9%) with a mean age of 52.68 years (SD = 10.44). Around one-fourth of the staff reported having chronic diseases, and 60% have ever been infected by COVID-19. All staff were vaccinated with at least one dose of the COVID-19 vaccine, and 87.4% of them received three doses or more.

Vaccine hesitancy among staff was low (mean = 2.45, SD = 0.82) (Table 3). Around 7% of the residents had a higher level of vaccine hesitancy (scored 4 or 4+ out of 7). Institutional mean scores of vaccine hesitancy ranged from 1.30 to 3.52.

### 3.3. Characteristics of Study RCHEs

All 31 study RCHEs were from the private sector (there were three only providing data of staff but not residents), with a mean facility size of 85.0 (range: 22–245). They were located throughout Hong Kong (Hong Kong Island: 7; Kowloon: 11; the New Territories: 13). Around one-fourth of them were government subsidized.

### 3.4. Predictors of Vaccine Hesitancy among Residents

Social loneliness (coefficient [b] = 0.081, standard error (SE) = 0.040, *p* = 0.047) and anxiety (b = 0.138, SE = 0.041, *p* = 0.001) positively predicted general vaccine hesitancy, while cognitive ability (b = −0.307, SE = 0.106, *p* = 0.004), the number of doses of COVID-19 vaccines taken (b = −0.312, SE = 0.115, *p* = 0.007), and the institutional vaccination rate negatively predicted general vaccine hesitancy (b = −0.629, SE = 0.252, *p* = 0.014) (Model 2 of Table 4).

For the respective 5C constructs of vaccine hesitancy, anxiety negatively predicted confidence (b = −0.143, SE = 0.055, *p* = 0.010, Model 2 of Table S1B). Institutional vaccination rate negatively predicted complacency (b = −1.085, SE = 0.500, *p* = 0.039, Model 2 of Table S1C). Age negatively predicted calculation (b = −0.026, SE = 0.013, *p* = 0.048, Model 2 of Table S1D). Age (b = −0.023, SE = 0.009, *p* = 0.009, Model 2 of Table S1E) and the number of COVID-19 doses taken (b = −0.395, SE = 0.148, *p* = 0.009) negatively predicted constraints, while anxiety positively predicted constraints (b = 0.221, SE = 0.050, *p* < 0.001). Age (b = 0.024, SE = 0.010, *p* = 0.021, Model 2 of Table S1F) and cognitive ability (b = 0.481, SE = 0.153, *p* = 0.002) positively predicted collective responsibility, while social loneliness (b = −0.126, SE = 0.058, *p* = 0.031) and anxiety (b = −0.133, SE = 0.059, *p* = 0.025) negatively predicted collective responsibility.

### 3.5. Predictors of Vaccine Hesitancy among Staff

Anxiety (b = 0.059, SE = 0.029, *p* = 0.044, Model 2 of Table 5) and social (b = 0.074, SE = 0.035, *p* = 0.040) and emotional loneliness (b = 0.097, SE = 0.034, *p* = 0.004) positively predicted vaccine hesitancy, while the number of doses of COVID-19 vaccines taken negatively predicted vaccine hesitancy (b = −0.339, SE = 0.141, *p* = 0.018). The size of the residential home was the only institutional factor that (positively) predicted vaccine hesitancy (b = 0.003, SE = 0.001, *p* = 0.002).

In the regression models using the respective 5C constructs as the outcomes, only social loneliness (b = −0.092, SE = 0.047, *p* = 0.054, Model 2 of Table S2B) and anxiety (b = −0.073, SE = 0.039, *p* = 0.065) marginally predicted confidence. Age (b = 0.024, SE = 0.012, *p* = 0.005) and emotional loneliness (b = 0.157, SE = 0.055, *p* = 0.044, Model 2 of Table S2C) positively predicted complacency, while the number of vaccines taken did the reverse (b = −0.804, SE = 0.234, *p* = 0.001). None of the variables predicted calculation (Table S2D). Emotional loneliness positively predicted constraints (b = 0.128, SE = 0.056, *p* = 0.025, Model 2 of Table S2E). Resident infection rate negatively predicted collective responsibility (b = −2.549, SE = 1.189, *p* = 0.039, Model 2 of Table S2F).

## 4. Discussion

### 4.1. Principal Findings

We conducted a cross-sectional survey with residents and staff in RCHEs using a clustered convenient sampling framework. Data regarding demographics, vaccine hesitancy, and psychological status were solicited from residents who passed the Mini-cog and staff. To our knowledge, this is the first study unraveling vaccine hesitancy with psychological status in this understudied setting. We found that vaccine hesitancy among residents and staff in RCHEs was low (residents: 2.44/7.00; staff: 2.45/7.00) shortly after region-wide explosive COVID-19 outbreaks. Anxiety and loneliness positivity predicted vaccine hesitancy among residents and staff. Specifically, social loneliness predicted residents’ vaccine hesitancy, while emotional loneliness predicted that of staff. Devising strategies to address hesitancy and potential delays in vaccination would be crucial for future infection prevention in RCHEs. Our results have five public health implications.

### 4.2. Result Implications

First, despite the low level of vaccine hesitancy reported in this study, its temporal changes in RCHEs need close monitoring. Our result may not be consistent with overseas findings in a comparable survey period, that a significant proportion (30.9%) of nurses in Greece were hesitant toward further COVID-19 vaccines [38]. Our study period (July–November 2022) was only three months after the peak of daily RCHE incidence in the primary Omicron wave [1]. The earlier finding that the heavier psychological impact induced by the pandemic lowered the level of vaccine hesitancy [39] probably explained our findings. Nevertheless, the COVID-19 pandemic is far from over in 2023 [40], and there is a call for preventing reinfection due to the further increase in disease burden [41], a longitudinal study tracking the changes of vaccine hesitancy in RCHEs is warranted.

Second, there should be financial and technical support to RCHEs to facilitate residents’ communications with their families and healthcare professionals in future outbreaks. Access to information is crucial to ease nerve-racking uncertainty. However, this could be challenging for residents in an isolated environment [42]. The information deprivation and lack of trustworthy individuals to rely on may contribute to vaccine hesitancy in residents. This association echoes the previous findings on young adults [18] and the general population [43]. Furthermore, the lack of social support may hamper trust in institutions [18]. With family members as important sources of information, frail residents may not understand the importance of vaccinating against COVID-19 to protect themselves and others, as we can see from the associations between social loneliness and collective responsibility. This may also explain our observation that residents with better cognitive ability were less hesitant. To reduce loneliness, active recreation activities in RCHEs are effective means, e.g., [44,45], which could be improved by virtual reality technology [46], assistive pet robots [47], and telephone outreach [48].

Third, public health professionals should keep track of the anxiety level of residents and staff, and devise appropriate health communication strategies accordingly. Studies have revealed the association between anxiety and vaccine hesitancy amid the COVID-19 pandemic, yet the directions of the association differ, which may reflect differences in the salient contexts that induced the anxiety. For example, a study observed a negative association between anxiety and vaccine hesitancy in the early stage of the pandemic [36], which may reflect the anxiety towards the uncertain progression of the pandemic, while the positive association in this study may imply the anxiety about vaccines. Alongside the involvement of behavioral healthcare workers to reduce vaccine hesitancy [49], there is growing support for using technologies, such as socially assistive robots [50] and AI-powered chatbots [51], to increase social engagement, and provide social companions for RCHE residents. Future research endeavors are needed to investigate the proper interventions that can mitigate the anxiety of the residents and staff, for example, by actively providing information disseminated by the government and facilitating social interactions with their relatives and friends outside the care homes through various online communication tools.

Fourth, the presence of vaccine-hesitant individuals in RCHEs highlights the need for setting-specific interventions to overcome vaccine hesitancy. Amid a generally low level of vaccine hesitancy, we reported the presence of hesitant persons (residents: 10%; staff: 7%) shortly after explosive outbreaks. Since social influence can propagate vaccine hesitancy within clusters [52], and the acts of others are an important reference for decision making in a congregate environment [53], targeted inventions should be tailored for RCHEs. Echoing our findings that emotional loneliness predicted vaccine hesitancy among staff, overseas counterparts considered “having someone like themselves vaccinated” to be motivating [23], and peer counseling was a significant strategy in boosting the uptake rate among staff [54].

Fifth, it is important to build resilience in RCHE staff and maintain their well-being. In addition to being deprived of social interactions because of the social distancing measures and higher chances of being quarantined under a high-exposure working environment, providing care activities amid the COVID-19 pandemic was highly stressful and imposed an immense emotional burden on the staff [55]. Lack of emotional support, which implied the lack of buffer for the psychological distress brought on by the pandemic, together with anxiety, predicted vaccine hesitancy of staff. Interventions on coping strategies and stress management have to be provided to healthcare workers to teach them better strategies for dealing with their emotions and stress [56].

### 4.3. Limitations

This study has several limitations. First, our assessment of vaccine hesitancy might be underestimated due to social desirability bias, especially for residents whose survey was completed by interviews. Although alternative methods (e.g., implicit measure, qualitative data) might provide a more objective measurement [22], their feasibility in RCHEs needs further examination. Second, we assume consistency in survey data collected across a few months. This survey was launched amid a period of very limited RCHE visitation, so the survey data could not be collected all on the same day. Third, we did not distinguish the hesitancy towards routine vaccines and novel vaccines, which might not be the same. Lastly, caution should be exercised when extrapolating our results, as convenient sampling may hamper its generalizability.

## 5. Conclusions

The devastation inflicted by COVID-19 in RCHEs has laid bare the loopholes in the current infection control strategies. Given that achieving herd immunity is unlikely to end the COVID-19 pandemic [57], addressing vaccine hesitancy in RCHEs becomes critical in reducing the healthcare burden during the COVID-19 endemic era, especially when there are emerging variants like BA.2.8.6 [58]. This study offers invaluable insights into the significance of loneliness in interventions targeting vaccine hesitancy. Future research should focus on outbreak-adapted strategies to bolster social interactions, including recreational activities through virtual platforms, enhancing technological accessibility, and creating a community atmosphere in RCHEs. Investing in the emotional well-being and resilience of RCHE staff remains pivotal.

## Figures and Tables

**Table 1:** Variables examined in the multi-level models.

Factors	Residents	Staff
Individual level		
Age	✓	✓
Biological sex	✓	✓
Care level	✓	✓
Mini-cog score	✓	
Number of doses of COVID-19 vaccines taken	✓	✓
Ever infected by COVID-19	✓	✓
Anxiety	✓	✓
Emotional loneliness	✓	✓
Social loneliness	✓	✓
Facility level		
Facility size	✓	✓
Infection rate of COVID-19 among residents	✓	✓
Uptake rate of COVID-19 vaccines (1 dose or more)	✓	✓

**Table 2:** Characteristics of study subjects.

Characteristics	Residents (N = 204)		Staff (N = 168)	
	*n*	%	*n*	%
Biological sex				
Male	134	65.69	12	7.14
Female	70	34.31	156	92.86
Age				
Below 55	13 ^a^	6.37	-	-
55–64	44	21.57	-	-
65–74	52	25.49	-	-
75–84	50	24.51	-	-
85 or above	45	22.06	-	-
Age				
18–34	-	-	7	4.17
35–44	-	-	26	15.48
45–54	-	-	60	35.71
55–64	-	-	55	32.74
65 or above	-	-	19	11.31
Missing	-	-	1	0.6
Presence of chronic diseases				
No	6	2.94	124	73.81
Yes	178	87.26	39	23.21
Missing	20	9.8	5	2.98
Presence of food or drug allergy				
No	173	84.8	144	85.71
Yes	15	7.35	22	13.1
Missing	16	7.84	2	1.19
Education level				
Primary or below	95	46.57	26	15.48
Secondary	70	34.31	120	71.43
Tertiary	-	-	20	11.9
Missing	39	19.12	2	1.19
Care level				
Low	87	42.65	-	-
Middle	62	30.39	-	-
High	45	22.06	-	-
Missing	10	4.9	-	-
COVID-19 infection (ever)				
Yes	174	85.29	102	60.71
No	30	14.71	66	39.29
Hospitalization due to COVID-19				
No	97	47.55	-	-
Yes	13	6.37	-	-
Missing	94	46.08	-	-
Number of COVID-19 vaccines received				
Zero	5	2.45	0	0
One	19	9.31	2	1.19
Two	73	35.78	19	11.31
Three	99	48.53	138	82.14
Four	8	3.92	9	5.36

^a^ Being disabled or having poor cognitive ability.

**Table 3:** Descriptive statistics of mental health and vaccine hesitancy variables.

Variables	Residents (N = 204)				Staff (N = 168)			
	Missing	Range	Score *	SD	Missing	Range	Score *	SD
Mental health								
Anxiety	6	0–10	2.54	(2.38)	11	0–21	3.04	(2.97)
Loneliness	6	0–12	3.57	(2.88)	10	0–22	8.79	(2.71)
Social loneliness	7	0–6	1.79	(2.15)	10	0–10	2.71	(2.28)
Emotional loneliness	6	0–6	1.78	(1.63)	10	0–12	6.08	(2.33)
Vaccine hesitancy								
Overall (3C)	0	1–7	2.44	(1.05)	0	1–7	2.45	(0.82)
Overall (5C)	0	1–7	2.64	(0.86)	0	1–7	2.87	(0.70)
Confidence	0	1–7	5.62	(1.42)	0	1–7	5.87	(1.04)
Complacency	2	1–7	2.95	(1.73)	1	1–7	3.15	(1.34)
Constraints	0	1–7	1.88	(1.30)	2	1–7	2.04	(1.28)
Calculation	1	1–7	4.03	(1.91)	2	1–7	5.02	(1.37)
Collective responsibility	0	1–7	6.03	(1.41)	2	1–7	5.91	(1.09)

* It is the total for mental health variables, and the mean for vaccine hesitancy variables.

**Table 4:** Multilevel regression analysis predicting residents’ overall vaccine hesitancy (3C), with the institution ID as the second-level factor.

Factors	Model 1 (N = 186 ^†^, RCHE = 26)					Model 2 (N = 150 ^†^, RCHE = 20)		
	b	SE	t	*p*	b	SE	t	*p*
Age	−0.009	0.006	−1.441	0.151	−0.009	0.007	−1.268	0.207
Female	−0.024	0.163	−0.146	0.884	0.121	0.194	0.622	0.535
Emotional loneliness	−0.043	0.053	−0.820	0.414	−0.020	0.061	−0.336	0.738
Social loneliness	0.061	0.035	1.759	0.080	0.081	0.040	2.007	0.047 *
Anxiety	0.135	0.036	3.735	0.000 ***	0.138	0.041	3.378	0.001 **
Mini-cog score	−0.222	0.090	−2.457	0.015 *	−0.307	0.106	−2.897	0.004 **
Number of COVID-19 vaccines received	−0.254	0.103	−2.476	0.014 *	−0.312	0.115	−2.725	0.007 **
COVID-19 infection (ever)	−0.143	0.226	−0.630	0.529	−0.212	0.247	−0.859	0.392
Care level	−0.054	0.097	−0.552	0.582	−0.189	0.110	−1.716	0.088
Size of the residential home					−0.001	0.001	−0.632	0.528
Resident infection rate					−0.683	0.787	−0.868	0.387
RCHE’s vaccination rate (with 1 dose or more)					−0.629	0.252	−2.493	0.014 *

* *p* < 0.05, ** *p* < 0.01, *** *p* < 0.001. **^†^** The sample sizes differ from the original sample size (N = 204) due to missing data in the controlled variables.

**Table 5:** Multilevel regression analysis predicting staff’s overall vaccine hesitancy (3C), with the institution ID as the second-level factor.

Factors	Model 1 (N = 156 ^†^, RCHE = 25)				Model 2 (N = 125 ^†^, RCHE = 18)			
	b	SE	t	*p*	b	SE	t	*p*
Age	0.000	0.006	0.056	0.955	0.002	0.007	0.301	0.764
Female	0.046	0.274	0.169	0.866	−0.141	0.308	−0.459	0.647
Emotional loneliness	0.062	0.030	2.094	0.038 *	0.097	0.034	2.909	0.004 **
Social loneliness	0.052	0.030	1.719	0.088	0.074	0.035	2.083	0.040 *
Anxiety	0.045	0.023	1.932	0.055	0.059	0.029	2.033	0.044 *
Number of COVID-19 vaccines received	−0.341	0.143	−2.387	0.018 *	−0.339	0.141	−2.400	0.018 *
COVID-19 infection (ever)	−0.029	0.135	−0.218	0.828	0.077	0.142	0.543	0.588
Size of the residential home					0.003	0.001	3.212	0.002 **
Resident infection rate					0.999	0.835	1.197	0.234
RCHE’s vaccination rate (with 1 dose or more)					−0.280	0.206	−1.357	0.177

* *p* < 0.05, ** *p* < 0.01. ^†^ The sample sizes differ from the original sample size (N = 168) due to missing data in the controlled variables.

## Data Availability

The data presented in this study are available on request from the corresponding author. The data are not publicly available due to privacy issues.

## Supplementary Materials

The following supporting information can be downloaded at: https://www.mdpi.com/article/10.3390/vaccines11111700/s1, Table S1A: Multilevel regression analysis predicting residents’ overall vaccine hesitancy (5C), with the institution ID as the second-level factor; Table S1B: Multilevel regression analysis predicting residents’ vaccine confidence, with the institution ID as the second-level factor; Table S1C: Multilevel regression analysis predicting residents’ vaccine complacency, with the institution ID as the second-level factor; Table S1D: Multilevel regression analysis predicting residents’ vaccine calculation, with the institution ID as the second-level factor; Table S1E: Multilevel regression analysis predicting residents’ vaccine constraints, with the institution ID as the second-level factor; Table S1F: Multilevel regression analysis predicting residents’ vaccine collective responsibility, with the institution ID as the second-level factor; Table S2A: Multilevel regression analysis predicting staff’s overall vaccine hesitancy (5C), with the institution ID as the second-level factor; Table S2B: Multilevel regression analysis predicting staff’s vaccine confidence, with the institution ID as the second-level factor; Table S2C: Multilevel regression analysis predicting staff’s vaccine complacency, with the institution ID as the second-level factor; Table S2D: Multilevel regression analysis predicting staff’s vaccine calculation, with the institution ID as the second-level factor; Table S2E: Multilevel regression analysis predicting staff’s vaccine constraints, with the institution ID as the second-level factor; Table S2F: Multilevel regression analysis predicting staff’s vaccine collective responsibility, with the institution ID as the second-level factor.
