# Supplementary material for: Revisiting Vaccine Hesitancy in Residential Care Homes for the Elderly for Pandemic Preparedness: A Lesson from COVID-19
Source: Vaccines (Basel). 2023 Nov 8;11(11):1700. doi: 10.3390/vaccines11111700 (PMC10675220; doi:10.3390/vaccines11111700)
Supplement: Supplementary file 1 [file vaccines-11-01700-s001.zip › vaccines-2665303-supplementary.pdf]

SUPPLEMENTARY MATERIALS

Table S1A. Multilevel regression analysis predicting residents' overall vaccine hesitancy (5C), with the institution ID as the second-level factor

	Model 1 (N = 186, RCHE = 26)				Model 2 (N = 150, RCHE = 20)			
	b	SE	t	p	b	SE	t	p
Age	-0.013	0.004	-2.924	0.004**	-0.015	0.005	-2.950	0.004**
Female	0.016	0.119	0.136	0.892	0.116	0.138	0.837	0.404
Emotional loneliness	-0.033	0.038	-0.882	0.379	-0.007	0.042	-0.159	0.874
Social loneliness	0.016	0.025	0.628	0.531	0.045	0.028	1.602	0.112
Anxiety	0.132	0.026	5.142	0.000***	0.136	0.028	4.792	0.000***
Mini-Cog score	-0.204	0.065	-3.127	0.002**	-0.225	0.075	-2.979	0.003**
Number of doses taken	-0.292	0.077	-3.788	0.000***	-0.318	0.086	-3.715	0.000***
Ever infected by COVID-19	-0.222	0.163	-1.364	0.174	-0.249	0.174	-1.433	0.154
Care level	-0.026	0.073	-0.357	0.722	-0.089	0.080	-1.107	0.270
Size of the residential home					0.000	0.001	-0.080	0.937
Resident infected rate					-0.616	0.618	-0.997	0.323
RCHE's vaccination rate (with 1 dose or more)					-0.402	0.224	-1.800	0.088.

. $p < 0.10$, *$p < 0.05$, **$p < 0.01$, ***$p < 0.001$.

Table S1B. Multilevel regression analysis predicting residents' vaccine confidence, with the institution ID as the second-level factor

	Model 1 (N = 186, RCHE = 26)				Model 2 (N = 150, RCHE = 20)			
	b	SE	t	p	b	SE	t	p
Age	0.002	0.009	0.242	0.809	-0.006	0.010	-0.668	0.505
Female	0.105	0.225	0.466	0.642	-0.314	0.262	-1.196	0.234
Emotional loneliness	0.110	0.074	1.483	0.140	0.110	0.082	1.333	0.185
Social loneliness	-0.046	0.049	-0.937	0.350	-0.068	0.054	-1.257	0.211
Anxiety	-0.161	0.051	-3.169	0.002**	-0.143	0.055	-2.597	0.010*
Mini-Cog score	0.039	0.127	0.307	0.759	0.135	0.143	0.942	0.348
Number of doses taken	0.257	0.138	1.862	0.064.	0.350	0.155	2.263	0.025*
Ever infected by COVID-19	0.327	0.317	1.033	0.303	0.624	0.333	1.870	0.064.
Care level	-0.066	0.131	-0.504	0.615	0.100	0.149	0.669	0.504
Size of the residential home					0.002	0.002	1.274	0.205
Resident infected rate					0.907	1.064	0.853	0.395
RCHE's vaccination rate (with 1 dose or more)					0.571	0.341	1.674	0.096.

. $p < 0.10$, *$p < 0.05$, **$p < 0.01$, ***$p < 0.001$.

Table S1C. Multilevel regression analysis predicting residents' vaccine complacency, with the institution ID as the second-level factor

	Model 1 (N = 184, RCHE = 26)				Model 2 (N = 148, RCHE = 20)			
	b	SE	t	p	b	SE	t	p
Age	-0.004	0.011	-0.345	0.730	-0.007	0.013	-0.510	0.611
Female	-0.158	0.286	-0.555	0.580	-0.291	0.352	-0.826	0.410
Emotional loneliness	-0.020	0.091	-0.219	0.827	0.046	0.108	0.424	0.672
Social loneliness	0.052	0.061	0.866	0.387	0.043	0.071	0.605	0.546
Anxiety	0.117	0.063	1.865	0.064.	0.133	0.072	1.840	0.068.
Mini-Cog score	-0.192	0.157	-1.222	0.223	-0.290	0.189	-1.532	0.128
Number of doses taken	-0.280	0.178	-1.574	0.118	-0.333	0.210	-1.583	0.116
Ever infected by COVID-19	-0.347	0.393	-0.884	0.378	-0.256	0.440	-0.582	0.562
Care level	-0.086	0.170	-0.505	0.614	-0.193	0.202	-0.956	0.341
Size of the residential home					0.002	0.003	0.624	0.541
Resident infected rate					-1.279	1.473	-0.868	0.388
RCHE's vaccination rate (with 1 dose or more)					-1.085	0.500	-2.170	0.039*

. $p < 0.10$, *$p < 0.05$, **$p < 0.01$, ***$p < 0.001$.

Table S1D. Multilevel regression analysis predicting residents' vaccine calculation, with the institution ID as the second-level factor

	Model 1 (N = 185, RCHE = 26)				Model 2 (N = 149, RCHE = 20)			
	b	SE	t	p	b	SE	t	p
Age	-0.021	0.011	-1.836	0.068.	-0.026	0.013	-1.998	0.048*
Female	0.071	0.305	0.232	0.817	0.223	0.357	0.627	0.532
Emotional loneliness	-0.001	0.097	-0.014	0.989	0.051	0.109	0.466	0.642
Social loneliness	-0.140	0.064	-2.174	0.031*	-0.100	0.073	-1.383	0.169
Anxiety	0.089	0.066	1.336	0.183	0.061	0.073	0.835	0.406
Mini-Cog score	-0.119	0.167	-0.717	0.475	-0.032	0.194	-0.165	0.870
Number of doses taken	-0.290	0.194	-1.496	0.137	-0.137	0.222	-0.615	0.540
Ever infected by COVID-19	-0.650	0.418	-1.554	0.122	-0.353	0.447	-0.788	0.432
Care level	0.138	0.183	0.754	0.452	0.158	0.207	0.762	0.448
Size of the residential home					0.001	0.003	0.371	0.716
Resident infected rate					-0.988	1.633	-0.605	0.548
RCHE's vaccination rate (with 1 dose or more)					-0.493	0.607	-0.811	0.428

. $p < 0.10$, *$p < 0.05$, **$p < 0.01$, ***$p < 0.001$.

Table S1E. Multilevel regression analysis predicting residents' vaccine constraints, with the institution ID as the second-level factor

	Model 1 (N = 185, RCHE = 26)				Model 2 (N = 150, RCHE = 20)			
	b	SE	t	p	b	SE	t	p
Age	-0.021	0.007	-2.938	0.004**	-0.023	0.009	-2.639	0.009**
Female	-0.004	0.187	-0.023	0.982	-0.015	0.240	-0.061	0.952
Emotional loneliness	-0.069	0.059	-1.168	0.244	-0.044	0.074	-0.592	0.555
Social loneliness	0.026	0.039	0.666	0.506	0.070	0.049	1.434	0.154
Anxiety	0.195	0.041	4.804	0.000***	0.221	0.050	4.460	0.000***
Mini-Cog score	-0.228	0.102	-2.225	0.027*	-0.203	0.131	-1.545	0.125
Number of doses taken	-0.356	0.119	-2.987	0.003**	-0.395	0.148	-2.669	0.009**
Ever infected by COVID-19	0.042	0.256	0.165	0.869	0.000	0.303	0.000	1.000
Care level	-0.014	0.113	-0.127	0.899	-0.061	0.139	-0.438	0.662
Size of the residential home					0.000	0.002	0.166	0.871
Resident infected rate					0.694	1.062	0.654	0.516
RCHE's vaccination rate (with 1 dose or more)					0.403	0.379	1.062	0.303

. $p < 0.10$, *$p < 0.05$, **$p < 0.01$, ***$p < 0.001$.

Table S1F. Multilevel regression analysis predicting residents' vaccine collective responsibility, with the institution ID as the second-level factor

	Model 1 (N = 186, RCHE = 26)				Model 2 (N = 150, RCHE = 20)			
	b	SE	t	p	b	SE	t	p
Age	0.019	0.008	2.346	0.020*	0.024	0.010	2.338	0.021*
Female	-0.161	0.221	-0.731	0.466	-0.296	0.281	-1.055	0.293
Emotional loneliness	0.005	0.071	0.069	0.945	-0.008	0.087	-0.095	0.924
Social loneliness	-0.076	0.047	-1.621	0.107	-0.126	0.058	-2.176	0.031*
Anxiety	-0.130	0.048	-2.684	0.008**	-0.133	0.059	-2.276	0.025*
Mini-Cog score	0.430	0.121	3.542	0.001***	0.481	0.153	3.137	0.002**
Number of doses taken	0.178	0.140	1.270	0.206	0.271	0.169	1.610	0.112
Ever infected by COVID-19	-0.301	0.304	-0.991	0.323	-0.199	0.356	-0.558	0.578
Care level	0.079	0.133	0.597	0.551	0.257	0.161	1.598	0.113
Size of the residential home					0.002	0.002	0.739	0.486
Resident infected rate					0.016	1.172	0.013	0.989
RCHE's vaccination rate (with 1 dose or more)					0.272	0.391	0.696	0.501

*. $p < 0.10$, *$p < 0.05$, **$p < 0.01$, ***$p < 0.001$.

Table S2A. Multilevel regression analysis predicting staff's overall vaccine hesitancy (5C), with the institution ID as the second-level factor

	Model 1 (N = 156, RCHE = 25)				Model 2 (N = 125, RCHE = 18)			
	b	SE	t	p	b	SE	t	p
Age	0.000	0.005	-0.018	0.986	0.003	0.006	0.456	0.649
Female	0.023	0.225	0.102	0.919	-0.148	0.258	-0.575	0.566
Emotional loneliness	0.078	0.024	3.225	0.002**	0.088	0.028	3.125	0.002**
Social loneliness	0.029	0.025	1.189	0.236	0.036	0.030	1.205	0.231
Anxiety	0.061	0.019	3.171	0.002**	0.071	0.024	2.907	0.004**
Number of vaccines uptaken	-0.247	0.117	-2.113	0.036*	-0.242	0.118	-2.044	0.043*
Ever infected by COVID-19	-0.027	0.110	-0.249	0.804	0.006	0.118	0.053	0.958
Size of the residential home					0.002	0.001	2.185	0.031*
Resident infected rate					1.033	0.699	1.479	0.142
RCHE's vaccination rate (with 1 dose or more)					-0.305	0.172	-1.767	0.080.

. $p < 0.10$, *$p < 0.05$, **$p < 0.01$, ***$p < 0.001$.

Table S2B. Multilevel regression analysis predicting staff's vaccine confidence, with the institution ID as the second-level factor

	Model 1 (N = 156, RCHE = 25)				Model 2 (N = 125, RCHE = 18)			
	b	SE	t	p	b	SE	t	p
Age	0.011	0.009	1.240	0.217	0.014	0.010	1.416	0.160
Female	0.029	0.368	0.078	0.938	-0.014	0.412	-0.034	0.973
Emotional loneliness	-0.044	0.040	-1.099	0.274	-0.043	0.045	-0.963	0.337
Social loneliness	-0.050	0.040	-1.247	0.214	-0.092	0.047	-1.951	0.054.
Anxiety	-0.045	0.031	-1.443	0.151	-0.073	0.039	-1.864	0.065.
Number of vaccines uptaken	0.055	0.190	0.291	0.771	0.048	0.190	0.253	0.801
Ever infected by COVID-19	-0.092	0.179	-0.511	0.610	-0.074	0.192	-0.386	0.700
Size of the residential home					-0.002	0.001	-1.362	0.262
Resident infected rate					-1.091	1.157	-0.943	0.354
RCHE's vaccination rate (with 1 dose or more)					0.030	0.296	0.101	0.922

. $p < 0.10$, *$p < 0.05$, **$p < 0.01$, ***$p < 0.001$.

Table S2C. Multilevel regression analysis predicting staff's vaccine complacency, with the institution ID as the second-level factor

	Model 1 (N = 156, RCHE = 25)				Model 2 (N = 125, RCHE = 18)			
	b	SE	t	p	b	SE	t	p
Age	0.016	0.011	1.520	0.131	0.024	0.012	2.012	0.047*
Female	0.141	0.453	0.311	0.757	-0.373	0.505	-0.740	0.461
Emotional loneliness	0.082	0.049	1.688	0.093.	0.157	0.055	2.856	0.005**
Social loneliness	0.057	0.050	1.144	0.254	0.033	0.058	0.578	0.565
Anxiety	0.058	0.039	1.502	0.135	0.058	0.048	1.212	0.228
Number of vaccines uptaken	-0.789	0.234	-3.377	0.001***	-0.804	0.234	-3.443	0.001***
Ever infected by COVID-19	-0.278	0.219	-1.266	0.208	0.032	0.236	0.136	0.892
Size of the residential home					0.004	0.002	2.503	0.064.
Resident infected rate					-0.542	1.420	-0.381	0.705
RCHE's vaccination rate (with 1 dose or more)					-0.606	0.364	-1.666	0.124

. $p < 0.10$, *$p < 0.05$, **$p < 0.01$, ***$p < 0.001$.

Table S2D. Multilevel regression analysis predicting staff's vaccine calculation, with the institution ID as the second-level factor

	Model 1 (N = 156, RCHE = 25)				Model 2 (N = 125, RCHE = 18)			
	b	SE	t	p	b	SE	t	p
Age	-0.002	0.011	-0.147	0.883	0.004	0.012	0.371	0.712
Female	-0.080	0.484	-0.166	0.869	0.074	0.506	0.147	0.884
Emotional loneliness	0.071	0.052	1.373	0.172	0.012	0.056	0.216	0.829
Social loneliness	-0.062	0.053	-1.164	0.246	-0.068	0.058	-1.160	0.249
Anxiety	0.081	0.041	1.956	0.052.	0.071	0.049	1.449	0.150
Number of vaccines uptaken	0.075	0.248	0.301	0.764	0.113	0.240	0.470	0.639
Ever infected by COVID-19	0.017	0.232	0.075	0.940	-0.255	0.246	-1.035	0.303
Size of the residential home					-0.003	0.002	-1.493	0.193
Resident infected rate					0.223	1.609	0.138	0.891
RCHE's vaccination rate (with 1 dose or more)					-0.370	0.446	-0.829	0.424

. $p < 0.10$, *$p < 0.05$, **$p < 0.01$, ***$p < 0.001$.

Table S2E. Multilevel regression analysis predicting staff's vaccine constraints, with the institution ID as the second-level factor

	Model 1 (N = 156, RCHE = 25)				Model 2 (N = 125, RCHE = 18)			
	b	SE	t	p	b	SE	t	p
Age	-0.002	0.010	-0.183	0.855	0.002	0.012	0.202	0.841
Female	-0.002	0.438	-0.005	0.996	-0.298	0.516	-0.577	0.565
Emotional loneliness	0.124	0.047	2.644	0.009**	0.128	0.056	2.278	0.025*
Social loneliness	0.060	0.048	1.261	0.209	0.022	0.059	0.366	0.715
Anxiety	0.091	0.037	2.441	0.016*	0.095	0.049	1.949	0.054.
Number of vaccines uptaken	-0.276	0.223	-1.241	0.217	-0.368	0.236	-1.558	0.122
Ever infected by COVID-19	-0.035	0.207	-0.167	0.867	-0.067	0.237	-0.285	0.776
Size of the residential home					0.002	0.001	1.690	0.094.
Resident infected rate					1.661	1.397	1.189	0.237
RCHE's vaccination rate (with 1 dose or more)					-0.454	0.345	-1.317	0.190

. $p < 0.10$, *$p < 0.05$, **$p < 0.01$, ***$p < 0.001$.

Table S2F. Multilevel regression analysis predicting staff's vaccine collective responsibility, with the institution ID as the second-level factor

	Model 1 (N = 156, RCHE = 25)				Model 2 (N = 125, RCHE = 18)			
	b	SE	t	p	b	SE	t	p
Age	0.004	0.009	0.465	0.643	0.005	0.010	0.474	0.636
Female	-0.010	0.377	-0.027	0.979	0.045	0.427	0.105	0.917
Emotional loneliness	-0.062	0.041	-1.521	0.130	-0.090	0.046	-1.943	0.055.
Social loneliness	-0.057	0.041	-1.370	0.173	-0.093	0.049	-1.890	0.061.
Anxiety	-0.031	0.032	-0.974	0.331	-0.044	0.041	-1.077	0.284
Number of vaccines uptaken	0.135	0.196	0.690	0.491	0.205	0.197	1.040	0.301
Ever infected by COVID-19	-0.115	0.185	-0.621	0.536	-0.099	0.199	-0.497	0.621
Size of the residential home					-0.003	0.001	-2.002	0.106
Resident infected rate					-2.549	1.189	-2.143	0.039*
RCHE's vaccination rate (with 1 dose or more)					0.230	0.302	0.760	0.461

. $p < 0.10$, *$p < 0.05$, **$p < 0.01$, ***$p < 0.001$.
